# Supervised Machine Learning Applied to Wearable Sensor Data Can Accurately Classify Functional Fitness Exercises Within a Continuous Workout

**DOI:** 10.3389/fbioe.2020.00664

**Published:** 2020-07-07

**Authors:** Ezio Preatoni, Stefano Nodari, Nicola Francesco Lopomo

**Affiliations:** ^1^Department for Health, University of Bath, Bath, United Kingdom; ^2^Dipartimento di Ingegneria dell'Informazione, Università degli Studi di Brescia, Brescia, Italy

**Keywords:** automatic classification, inertial measurement unit, sport, on-field testing, activity monitoring, machine learning, wearable sensors

## Abstract

Observing, classifying and assessing human movements is important in many applied fields, including human-computer interface, clinical assessment, activity monitoring and sports performance. The redundancy of options in planning and implementing motor programmes, the inter- and intra-individual variability in movement execution, and the time-continuous, high-dimensional nature of motion data make segmenting sequential movements into a smaller set of discrete classes of actions non-trivial. We aimed to develop and validate a method for the automatic classification of four popular functional fitness drills, which are commonly performed in current circuit training routines. Five inertial measurement units were located on the upper and lower limb, and on the trunk of fourteen participants. Positions were chosen by keeping into account the dynamics of the movement and the positions where commercially-available smart technologies are typically secured. Accelerations and angular velocities were acquired continuously from the units and used to train and test different supervised learning models, including k-Nearest Neighbors (kNN) and support-vector machine (SVM) algorithms. The use of different kernel functions, as well as different strategies to segment continuous inertial data were explored. Classification performance was assessed from both the training dataset (k-fold cross-validation), and a test dataset (leave-one-subject-out validation). Classification from different subsets of the measurement units was also evaluated (1-sensor and 2-sensor data). SVM with a cubic kernel and fed with data from 600 ms windows with a 10% overlap gave the best classification performances, yielding to an overall accuracy of 97.8%. This approach did not misclassify any functional fitness movement for another, but confused relatively frequently (2.8–18.9%) a fitness movement phase with the transition between subsequent repetitions of the same task or different drills. Among 1-sensor configurations, the upper arm achieved the best classification performance (96.4% accuracy), whereas combining the upper arm and the thigh sensors obtained the highest level of accuracy (97.6%) from 2-sensors movement tracking. We found that supervised learning can successfully classify complex sequential movements such as those of functional fitness workouts. Our approach, which could exploit technologies currently available in the consumer market, demonstrated exciting potential for future on-field applications including unstructured training.

## Introduction

The problem of tracking, identifying and classifying human actions has received increasing interest over the years, as it plays a key role in many applied contexts, such as human-computer interface (Popoola and Wang, 2012; Sarig Bahat et al., 2015; Quitadamo et al., 2017; Bachmann et al., 2018), daily-life activity monitoring (Mannini and Sabatini, 2010; Cheng et al., 2015; Chetty and White, 2016), clinical assessment (Rawashdeh et al., 2016; Arifoglu and Bouchachia, 2017; Howell et al., 2017) and sports performance (Attal et al., 2015; Ghazali et al., 2018; Hsu et al., 2018). The development of unobtrusive technologies for motion capture (e.g., wearable inertial measurement units—IMUs), their widespread integration in relatively cheap, commercially available devices (e.g., smartphones, watches, activity trackers, heart rate monitors, sensorized insoles), and the push toward healthier, more active life styles, have generated a multitude of existing and potential applications where automatic movement classification and assessment is fundamental (Attal et al., 2015; Cheng et al., 2015; Cust et al., 2019).

Sport coaching and training still largely rely on visual observation and subjective feedback, and they could benefit from quantitative input supporting decision making. Having quantitative real-time information about the amount, quality and intensity of the work carried out may play an important role at multiple levels. It could inform coaching and strength & conditioning planning, help monitoring training load, and evaluating the quality of movement performance (i.e., the outcome achieved) and movement execution (i.e., technique). It could also help improving injury prevention, as continuous monitoring could enable systematic screening of movement behavior, help identifying risk factors and mechanisms of injury, and support decision making in terms of pre- and rehabilitation programmes (Jones and Wallace, 2005).

Motion capture has traditionally relied on optical-based solutions, but recent development in microelectronics has generated increased interest and research efforts into wearable technologies (Adesida et al., 2019). Wearable systems are particularly suitable to sport-specific needs (van der Kruk and Reijne, 2018), since: (1) sport usually takes place in uncontrolled and unstructured settings, with environmental conditions difficult to be predicted *a priori* (e.g., weather, interaction with equipment and other people) and many possible measurement interferences (e.g., electromagnetic noise); (2) the size of the acquisition volume inherently depends on the type of practiced sport (e.g., team vs. individual, indoor vs. outdoor); (3) sensors used to capture sports movements should be both robust and non-obtrusive for the athlete (i.e., ecologically transparent). Systems based on wearable devices, including low-cost activity trackers, smartwatches and smartphones (Ahmad et al., 2017), have kept evolving and are widely available for the consumer market, including clinical uses and sports applications (Ghazali et al., 2018; Hsu et al., 2018). Wearable technologies for motion analysis are predominantly inertial measurement units (IMUs) (Davila et al., 2017), which, thanks to their low cost and minimal obtrusiveness, represent an optimal solution for tracking and assessing sports movement on-field (Hsu et al., 2018; van der Kruk and Reijne, 2018; Adesida et al., 2019).

Despite the widespread of wearable technology in both applied and research environments, the use of wearable data as input of algorithms for the detection and classification of human actions remains non-trivial, especially in sport. Indeed, sport activities typically involve a large variety of movements, execution technique demonstrates inherent inter- and intra-individual variability, and data is of high-dimensionality (Endres et al., 2012; Hsu et al., 2018). For this reason, no “one-size-fits-all” approach exists (Crema et al., 2019), and bespoke solutions have been reported to address only specific needs, including: recognition/classification (i.e., “what type” of task a subject performs) or identification of the achieved performance (i.e., “how good” the subject performs the task, with respect to a specific reference). In this perspective, the literature has focused the analysis on very specific sport activities and tasks (Cust et al., 2019).

Among fitness activities, functional training combines aerobic conditioning, weightlifting, interval training, balancing, gymnastics, and functional fitness movements (i.e., exercises that mimic daily life requirements, such as lifting weights) performed at high level of intensity (Liebenson, 2006). Functional fitness has been shown to improve cardiovascular capacity, muscle tone and central nervous system efficiency (Barbieri et al., 2019; Singh and Saini, 2019), but may also increase risk of musculoskeletal injuries affecting shoulder, lower back and knee joints (Gianzina and Kassotaki, 2019). It is therefore important to provide athletes with reliable feedback about their efforts, and guide them toward safe movement technique. The availability of a quantitative system for the monitoring of movement completion and overall performance would aid coaching and judging. Functional fitness workouts often consist of continuous sequences of movements, and the identification and assessment of individual elements within the sequence currently relies on visual observation and the expertise of the coach. The wide spectrum of situations in terms of dynamics and body part involved represents a difficult challenge for the automatic classifications of activities and makes it a good proof of concept for the scope of our study. Being able to identify specific movements within a complex movement sequence could be the starting point of a number of useful applications such as counting the number of movement tasks completed, and hence assessing technique and training load for both performance and injury prevention purposes.

We aimed to develop and validate a bespoke algorithm for the automatic recognition and classification of four popular functional fitness drills, when performed in a continuous workout. In particular, we wanted to test the capability of supervised machine learning approaches when fed with data from a network of five wearable inertial sensors on the body. Also, we carried out a sensitivity analysis, which could indicate whether subsets of the available measuring units could still provide acceptable classification performance.

## Materials and Methods

### Population

Fourteen healthy participants (11 males and three females, age 18–50) with at least 6-months experience in functional training activities volunteered to take part in this study. All participants were physically active, free from any neurological disease and musculoskeletal condition at the time of testing, and familiar with the movement tasks to be performed. The study protocol received ethical approval by the local research ethics committee (reference number EP 17/18 247). Volunteers were informed about experimental procedures and signed informed consent before participating. Based on the existing literature (Cust et al., 2019) and the exploratory nature of the study, a sample size >12 was deemed adequate to address the research objectives.

### Experimental Setup

**Figure 1:**
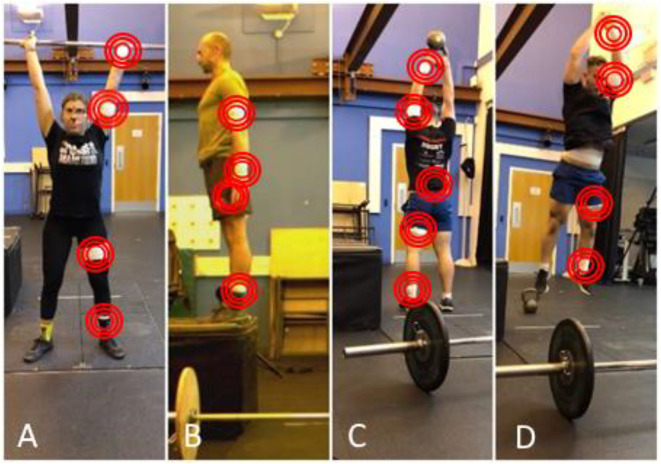
Experimental setup and movement tasks, where the position of IMU sensors has been highlighted. **(A)** “Clean and Jerk,” **(B)** “Box Jump,” **(C)** “American Swing,” and **(D)** “Burpee”. All the five sensors were worn by the participants throughout the execution of the protocol.

Five wearable units (Trigno Avanti Wireless EMG System, Delsys Inc., USA) were secured to the participants via double-sided hypoallergenic tape and elastic straps. IMUs were located onto specific anatomical landmarks (Figure 1), which included the left ankle, thigh, upper arm and wrist, and trunk (L5-S1 level). These positions were chosen to: (a) reproduce the locations where commercially available devices with embedded motion monitors (e.g., smart watches, smart phones, shoe-sensors) could be positioned; and, (b) to capture whole body information and drill dynamics whilst allowing the natural execution of movements, avoiding obstruction or discomfort for the subject. The wearable units embed tri-axial accelerometers, gyroscopes and magnetometers and were able to synchronously communicate with the system base station via Bluetooth Low Energy (BLE) wireless protocol ensuring an acquisition rate of 148.15 Hz.

Accelerations and angular velocities (±16 g, ±2,000°/s) were acquired continuously throughout the workout by means of the wearable units; magnetometer measurements were excluded due to the presence of ferromagnetic materials, very close to the acquisition volume. Data coming from the sensors were synchronized with a commercial video-camera (Oqus Video 210c, Qualisys AB, Sweden; 50 Hz) via a dedicated trigger module (Trigger Module, Delsys Inc. USA) connected to both systems. The video camera was positioned in front of workout station, thus allowing the correct acquisition of all the performed movements.

### Experimental Protocol

**Figure 2:**
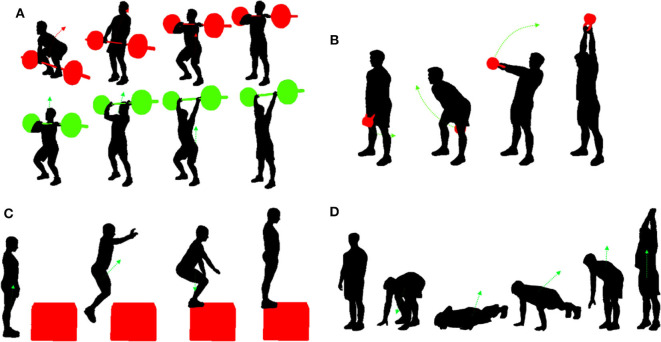
Schematic representation of the execution stages of the fitness training drills used in this study: **(A)** “Clean and Jerk,” **(B)** “American Swing,” **(C)** “Box Jump,” and **(D)** “Burpee”.

Participants were asked to execute a workout session including four popular functional training drills (Figure 2). These consisted of:

- “*Clean and Jerk*” (*C*&*J*). A weighted barbell is lifted from the ground to over the head in two subsequent movements: the “clean,” where the barbell is pulled from the floor to a racked position across the shoulders, and “jerk,” where the barbell is raised above the head, and a stable position is achieved by keeping straight legs and arms, and feet, torso and barbell lie in the same plane.
- “*American Swing” (AS*). A kettlebell is grasped with both hands and swung from below the groin to above the head, keeping the arms straight. The upward momentum of the kettlebell is predominantly generated by the explosive extension of the hip.
- “*Box Jump” (BJ)*. The participant start from a standing position in front of a box, performs a countermovement jump to land on top of it, achieves a stable upright position, and completes the task by returning to the start position.
- “*Burpee” (BP)*. A four-stage exercise, where the participant starts from a standing position, squats placing the hands on the floor, kicks back into a plank position while keeping the arms extended, returns in the squat position and, jumps up extending the upper limbs overhead.

All the movement tasks were illustrated to the participants at the start of the session, following the standards approved for competition (CrossFit, 2019; WODstar, 2019). A 50 cm box was used in the Box Jump exercise for all participants, whereas drills with an added resistance were differentiated between female and male participants, and set to, respectively: 20 and 40 kg in the Clean & Jerk; 12 and 16 kg in the American Swing.

After a self-directed warm up, and some repetitions to familiarize with the experimental setup, each participant performed 3 sets of functional fitness activities structured as follows:

Set 1 (classifier training dataset):

- 3 × C&J + 3 × BJ + 3 × AS + 3 × BP

Set 2 (workout simulation session, classifier test dataset):

- *1st Round:* 1 × C&J + 1 × BJ + 1 × AS + 1 × BP
- *2nd Round:* 2 × C&J + 2 × BJ + 2 × AS + 2 × BP
- *3rd Round:* 3 × C&J + 3 × BJ + 3 × AS + 3 × BP
- *4th Round:* 4 × C&J + 4 × BJ + 4 × AS + 4 × BP

Set 3 (classifier training dataset):

- 3 × C&J + 3 × BJ + 3 × AS + 3 × BP

Five-minute recovery was allowed between sets, whereas movements were executed sequentially with no rest allowed between repetitions of the same exercise, different exercises or rounds. This was done to ensure ecological validity with respect to a real functional fitness training session, and to challenge the capability of the classifying algorithm to recognize movements when they are performed without clear breaks in-between them. The order of movement execution was randomized between participants, to avoid possible bias due to repetitive patterns.

The workout simulation (Set 2) was preceded (Set 1) and followed (Set 3) by a sequence of three repetitions of each task. The pre- and post-workout session were used as training sets for the machine learning algorithms, and were both included so that the classification method could be robust to the possible changes in movement execution caused by fatigue or learning effects in the participants.

### Data Analysis

The three components of acceleration and angular velocity from the five IMUs (6 × 5 = 30 continuous timeseries) were used as input data for the classifying algorithm. Kinematic quantities were not filtered, and frequency-domain signals were attained through transforming the time-domain signals via Fast Fourier Transform. Data features in the time and frequency domain (Table 1) were extracted from data windows moving across the original kinematics timeseries. This process aimed to reduce the signals into distinctive characteristics of specific movement tasks or part of them. The more each movement can be separated in feature space, the higher the achieved recognition and classification performance is Zhang and Sawchuk (2013) and Hoettinger et al. (2016).

**Table 1:** Features extracted from each time window of each signal collected (from accelerometers and gyroscopes), and then used as input of the classification algorithm.

**Time domain**	**Frequency domain**
Mean value (Magnitude)	Mean value
Standard deviation	Power
Root mean square	Higher frequency
Mean absolute deviation	Lower frequency
Max value	Median frequency
Min value	Mean frequency
Kurtosis	Spectral entropy
Skewness	
Quartile (25th, 50th, 75th)	

**Figure 3:**
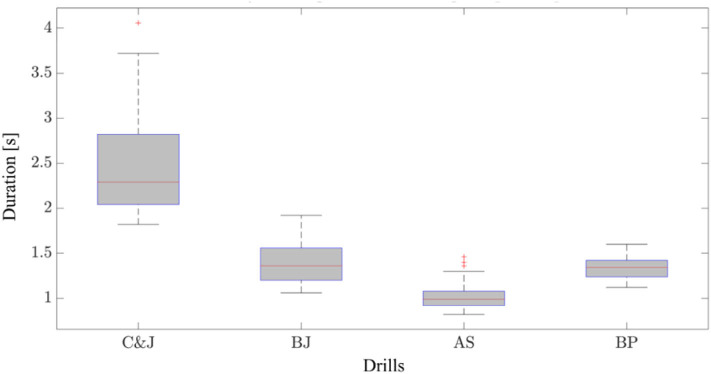
Distribution of individual movement durations in the analyzed population. C&J, Clean and Jerk; AS, American Swing; BJ, Box Jump; BP, Burpee.

To set suitable ranges for window duration and decide the amount of window overlap, we analyzed the distribution of movement durations across the population (Figure 3). Values between 300 and 600 ms (in increments of 100 ms) for window length, and of 0, 10, and 20% for the amount of overlap were chosen to study the sensitivity of the classification to the choice of windowing parameters. This allowed to have at least three time windows covering the execution of each movement or the transitions between subsequent movements. A [N × 540] feature matrix was generated for each participant, where N indicates the number of time windows in each session, and 540 is the overall number of features included in the analysis (5 sensors × 2 kinematic quantities per sensor × 3 directions per quantity ×18 features per quantity).

#### Data Labeling

A supervised approach to automatic classification was adopted, with video-based classification used as the gold standard for labeling each data window as a transition phase or as a part of one of the four possible functional fitness movements (Figure 2). Camera footage was used to identify the start and end of each movement and for their classification (i.e., labeling), as required by the supervised learning model. Movement recognition, timing and labeling were visually carried out by a single expert operator using freeware video editing software (VirtualDub, virtualdub.org). When a window spanned between a transition phase and one of the four movement tasks, a “majory” criterion was used. This implied assigning a movement label (i.e., “C&J,” “AS,” “BJ,” or “BP”) to a window where the movement covered more than 66% of its length. Otherwise, the transition label (i.e., “TRANS”) was allocated.

#### Classifier Training

After the extraction of the features and the labeling of associated windows, we trained different type of automatic classifiers using data Set 1 and 3. k-Nearest Neighbors (kNN), with different types of metrics (Euclidean, cosine, cubic or weighted distance) and number of neighbors (fine, k = 1; medium, k = 10; and coarse, k = 100), and Support Vector Machine (SVM) with several types of kernel functions (i.e., linear, quadratic, cubic and fine-medium-coarse Gaussian), were selected as the classifying algorithms to be tested. This choice was driven by the existing literature in the area of machine learning approaches addressing human motion (Camomilla et al., 2018; Cust et al., 2019) and sport (Cust et al., 2019) classification. At this stage, all the reported features (Table 1) were used to train the models.

#### Classifier Assessment

Two levels of classifier evaluation were carried out. Firstly (Stage 1), we performed a 5-fold cross-validation on the classifier training dataset (N = 14 participants, Set 1 and 3); this approach was used to mitigate the risk of overfitting by partitioning the dataset into k-folds and estimating the accuracy of each fold (Taha et al., 2018). We used this stage to select the most promising algorithm amongst the many tested. Finally (Stage 2), assessed the classifier performance on new data (i.e., the workout simulation dataset, Set 2) in a Leave-On-Subject-Out (LOSO) fashion (Hagenbuchner et al., 2015; Willetts et al., 2018). In this stage, the classifier was trained with data from Set 1 and 3 (including N-1 participants), and validated against data of the N-th participant, from Set 2; the N-th participant was iteratively changed, and results were reported averaging the multiple iterations. This approach guaranteed having independent data, in terms of both trials and individuals, between training and testing sets.

Classification accuracy (Equation 1) was evaluated as follows (Hoettinger et al., 2016; Davila et al., 2017):

(1) $$\begin{array}{l} Accuracy=\frac{TP+TN}{TP+TN+FP+FN} \end{array}$$

where TP, TN, FP and FN represent True Positive, True Negative and False Positive, respectively.

Once the optimal classifier was identified, the corresponding confusion matrix and Receiver Operating Characteristic (ROC) curves (in a multi-label “one-vs.-rest” assessment) were analyzed to assess the ability of the algorithm to recognize and correctly classify each functional fitness exercise. From the confusion matrix, for each exercise, we evaluated:

- the Positive Predictive Value (PPV), representing the precision of the classifier (Equation 2):

(2) $$\begin{array}{l} PPV=\frac{TP}{TP+FP} \end{array}$$

- the True Positive Rate (TPR), representing the sensitivity (also called recall) of the classifier (Equation 3):

(3) $$\begin{array}{l} TPR=\frac{TP}{TP+FN} \end{array}$$

#### Sensitivity Analysis

Two type of sensitivity analysis were carried out: (a) the effect of window length and overlapping, where all classifier types and all the features were included; and, (b) the effect of selecting a subset of the five available IMUs, which was analyzed starting from the classifier previously identified as giving the best outcome performance (as highlighted in validation Stage 1). For (b), the analysis was carried out starting from the data provided by each sensor in isolation and by considering data from pairs of sensors, as follows:

- wrist and ankle;
- wrist and lumbar area;
- wrist and thigh;
- wrist and upper arm;
- upper arm and ankle;
- upper arm and lumbar area;
- upper arm and thigh.

#### Feature Selection Analysis

Once the best performing subset of the five measurement units was identified, an exploratory analysis of the most significant features extracted was carried out. We used the minimum Redundancy Maximum Relevance (mRMR) filter-based algorithm applied to the standardized feature matrix, due to its trade-off between performance and efficiency (Peng et al., 2005; Wang et al., 2016). To compare the overall accuracy, a fixed number of features was identified starting from the analysis of the predictor importance scores performed on the training dataset; these features were then used to train the models and to test them following Stage 2 validation.

Training of the supervised learning models and analysis of classification performance were carried out through the Statistics and Machine Learning Toolbox and bespoke functions developed in Matlab (v R2019b, The Mathworks Inc.).

## Results

### k-Fold Cross-Validation of Classifier Performance and Sensitivity Analysis: Time Window and Overlap Parameters

When data input included all the five available sensors, both SVM- and kNN-type classifiers achieved good level of overall accuracy (Tables 2, 3, respectively). Accuracy ranged from 82.5% (SVM classifier with fine gaussian kernel, and 300 ms−10% overlap windows) to 97.8% (cubic kernel SVM classifier, with 600 ms−10% overlap windows).

**Table 2:** Overall classification performance (accuracy, in %) for Support Vector Machine (SVM) algorithms, as a factor of different kernel functions, window lengths, and percentage of window overlap.

		**Overlap [%]**			**Overlap [%]**			**Overlap [%]**		
		**0**	**10**	**20**	**0**	**10**	**20**	**0**	**10**	**20**
		**Linear**			**Quadratic**			**Cubic**		
Window [ms]	300	95.6	95.5	95.7	96.7	96.7	96.8	97.1	97.0	97.3
	400	95.7	96.0	96.2	97.2	97.1	97.1	97.3	97.3	97.3
	500	96.3	96.6	96.4	97.1	97.1	97.1	97.4	97.4	97.2
	600	96.2	97.0	96.4	97.0	97.7	97.7	97.0	97.8	97.7
		**Fine gaussian**			**Medium gaussian**			**Coarse gaussian**		
Window [ms]	300	82.6	82.5	82.7	95.5	95.5	95.7	93.5	93.7	94.0
	400	82.8	82.9	82.8	95.4	95.6	96.0	93.8	94.0	94.0
	500	83.6	83.2	83.3	95.2	95.4	95.5	93.6	94.6	94.3
	600	83.6	83.7	83.4	95.2	95.8	95.5	93.6	94.6	94.2

*Data from all the five IMUs available were used as input. Green bold numbers = best performance; red bold numbers = worst performance*.

**Table 3:** Overall classification performance (accuracy, in %) for k-Nearest Neighbors (kNN) algorithms, as a factor of different kernel functions, window lengths and percentage of window overlap.

		**Overlap [%]**			**Overlap [%]**			**Overlap [%]**		
		**0**	**10**	**20**	**0**	**10**	**20**	**0**	**10**	**20**
		**Fine Class**			**Medium Class**			**Coarse Class**		
Window [ms]	300	96.4	96.4	96.7	96.3	96.0	96.5	89.2	89.8	90.6
	400	96.3	96.3	97.0	96.1	96.5	96.8	89.0	90.1	90.5
	500	96.1	97.0	97.0	96.0	96.5	96.1	89.7	89.8	90.4
	600	96.2	97.2	96.7	95.7	96.4	96.2	89.0	89.9	90.5
		**Cosine**			**Cubic**			**Weighted**		
Window [ms]	300	96.4	96.3	96.6	94.0	93.7	94.1	96.4	96.3	96.8
	400	96.3	96.5	96.5	93.9	94.2	94.9	96.4	96.7	96.9
	500	96.1	96.7	96.3	94.6	94.7	94.8	96.3	96.6	96.4
	600	96.1	97.2	96.5	94.7	94.9	94.8	96.3	96.9	96.5

*Data from all the five IMUs available were used as input. Green bold numbers = best performance; red bold numbers = worst performance*.

### Testing SVM Performance With Training and Test Datasets

Considering the overall accuracy, the training time (a ratio of more than 20 between the slowest and the fastest classifier) and the computational costs (a ratio of more than 80 between the fastest and slowest classifiers, in terms of prediction speed), the SVM with cubic kernel applied to 600 ms−10% overlap windows appeared as the optimal learning model. The confusion matrix for this classifier (Table 4) showed that the trained model yielded to almost no (validation Stage 1) or few (validation Stage 2) misclassifications between different functional fitness movements. Specific accuracy ranged from 99.7% for burpees in the 5-fold cross-validation to 94.3% for the transition phase when tested on new data. All but one erroneous classification in the 5-fold cross-validation were from movement tasks identified as transition phases (64, 1.6% of the total) and, less frequently, from transitions confused for functional fitness drills (19, 0.5%). We had up to 18.9% of false negative rates in the AS drill, which reported the lowest level of precision (93.0%) and sensitivity (81.1%) (Table 5). Similar outcomes, but with lower percentage values, were reported by the LOSO validation on the test dataset. Precision and sensitivity values were always highest in the transition movements (94.9 and 97.8%, respectively), whereas the Clean & Jerk (89.3 and 82.2%) and American Swing (93.0 and 79.3%) showed the lowest performance results (Table 5).

**Table 4:** Confusion matrixes for the cubic kernel SVM algorithm with a 600 ms window length and 10% overlap.

**Stage 1: 5-fold Cross-Validation – Training Dataset**						
		**Predicted class**				
		**C&J**	**AS**	**BJ**	**BP**	**TRANS**
True class	C&J	228 (5.9%)				22 (0.6%)
	AS		107 (2.8%)			25 (0.6%)
	BJ			92 (2.4%)		13 (0.3%)
	BP		1 (0.0%)		138 (3.6%)	4 (0.1%)
	TRANS	2 (0.1%)	7 (0.2%)	5 (0.1%)	5 (0.1%)	3215 (83.2%)
**Stage 2: LOSO – Test Dataset**						
		**Predicted class**				
		**C&J**	**AS**	**BJ**	**BP**	**TRANS**
True class	C&J	434 (8.1%)	4 (0.1%)			90 (1.7%)
	AS	3 (0.1%)	214 (4.0%)		2 (0.0%)	51 (0.9%)
	BJ			174 (3.2%)		29 (0.5%)
	BP	2 (0.0%)			280 (5.2%)	43 (0.8%)
	TRANS	47 (0.9%)	12 (0.2%)	12 (0.2%)	20 (0.4%)	3961 (73.3%)

*Classification performance is reported from the two stages of validation as total counts and % of total. Blank cells correspond to a count of zero. LOSO = “leave one subject out.” Training = sensor data from movements of Set 1 and Set 3 of the experimental protocol, used to train the classifier. Test = sensor data from movements of Set 2 of the experimental protocol, not used to train the algorithm. Results are the average across multiple iterations. Green = correct prediction, red = misclassification*.

**Table 5:** Accuracy (ACC), precision (PPV, Positive Predictive Value) and sensitivity (TPR, True Positive Rate) for each functional fitness movement, related to the cubic kernel SVM algorithm with a 600 ms length windows and 10% overlap.

	**ACC**		**PPV**		**TPR**	
	**Stage 1**	**Stage 2**	**Stage 1**	**Stage 2**	**Stage 1**	**Stage 2**
C&J	99.4	97.3	99.1	89.3	91.2	82.2
AS	99.1	98.7	93.0	93.0	81.1	79.3
BJ	99.5	99.2	94.8	93.5	87.6	85.7
BP	99.7	98.8	96.5	92.7	96.5	86.2
TRANS	97.9	94.3	98.0	94.9	99.4	97.8

*Data from all the five IMUs available were used as input. Classification performance is reported from the two stages of validation as %, and results are the average across multiple iterations. Stage 1= 5-fold Cross-Validation—Training Dataset; Stage 2 = “leave one subject out” (LOSO) on test dataset. Green bold numbers = best performance; red bold numbers = worst performance*.

The analysis of ROC curves gives us the power of our classifier in a multi-label classification problem, as a function of the Type I error (i.e., 1—specificity), as it was a binary predictor. Considering the validation stages, the selected SVM classifier showed an almost null value for FPR in each functional fitness movement (<1%), with the TPR ranging from 84% (BJ classification) to 96% (BP classification). The highest value of TPR was reached in the classification of transition phases (99%), although, in TRANS the classifier also reported the highest level of FPR (7%). Finally, the Area Under the Curve (AUC), which describes the capability of the supervised learning model to distinguish between one class and the others, ranged between 0.98 and 1, therefore showing good overall classification performances.

### Sensitivity Analysis: Number of Sensors

When considering the data coming from a single sensor, the selected SVM classifier achieved good values of recognition rates in most cases (Table 6), with an overall accuracy between 83.2% (data from the ankle sensor, validation Stage 2) and 96.4% (data from the upper arm sensor, cross-fold validation). Using input data from pairs of IMUs generally improved the overall classification accuracy, pushing it up of several percentage point when testing on new data (Stage 2: from 83.2−91.0% to 92.0−93.0%). However, using two sensors did not match the performances obtained when data from all the sensors were utilized (93.0 vs. 97.8%).

**Table 6:** Overall classification performance (accuracy, in %) for Support Vector Machine (SVM) algorithms, with 600 ms−10% overlap windows.

**Validation**	**W**	**UA**	**T**	**A**	**L**		**W+A**	**W+L**	**W+T**	**W+UA**	**UA+A**	**UA+L**	**UA+T**
Stage 1	94.5	96.4	93.5	92.4	93.5		96.4	96.5	97.0	96.8	96.8	97.4	97.6
Stage 2	89.3	91.0	86.8	83.2	87.5		92.0	92.0	92.6	92.2	92.2	93.0	93.0

*Data from individual or pairs of IMUs were used as input. Stage 1= 5-fold Cross-Validation—Training dataset; Stage 2 = “leave one subject out” (LOSO) on workout dataset. Green bold numbers = best performance; red bold numbers = worst performance. W, Wrist; UA, Upper Arm; T, Thigh; A, Ankle; L, Lumbar Segment*.

In relation to the contribution of each sensor to the correct classification of individual functional drills, including data from the sensor placed on the upper limb (upper arm or wrist), or from a combination of a sensor on the upper limb and a sensor on the lumbar area or thigh, seemed to improve classifier performance, in at least 3 out of 4 movements and in the transition phases (Tables 7, 8). Only in the AS, the classifier seemed to perform relatively better when using data from the sensor placed on the lumbar spine (single sensor configuration). The worst overall performance was obtained when considering the data acquired by the only sensor placed on the ankle. Only for the AS, the algorithm did worse considering the data registered by the sensor placed on the wrist.

**Table 7:** Accuracy (ACC), precision (PPV, Positive Predictive Value) and sensitivity (TPR, True Positive Rate) for each functional fitness movement, related to the cubic kernel SVM algorithm with a 600 ms length windows and 10% overlap.

	**W**			**UA**			**T**			**A**			**L**		
	**ACC**	**PPV**	**TPR**	**ACC**	**PPV**	**TPR**	**ACC**	**PPV**	**TPR**	**ACC**	**PPV**	**TPR**	**ACC**	**PPV**	**TPR**
C&J	96.4	85.7	75.9	96.2	86.7	72.9	93.0	65.1	62.9	90.9	53.6	55.7	93.2	67.6	58.5
AS	96.8	81.3	48.1	97.3	78.5	63.7	97.5	81.7	65.9	97.6	82.6	65.2	97.8	82.1	71.1
BJ	98.6	83.9	76.8	99.1	90.9	83.7	96.9	61.3	45.3	95.4	36.5	28.1	98.1	87.9	57.1
BP	97.0	88.3	58.2	97.9	83.6	81.5	97.5	85.3	69.8	97.1	79.5	69.2	97.0	78.9	68.0
TRANS	89.7	90.2	96.8	91.5	92.7	96.3	88.6	90.6	94.7	85.3	89.0	91.8	89.0	90.5	95.5

*Data from individual IMUs were used as input. Classification performance is reported as %. For sake of clarity, only results from the most stringent validation (Stage 2 = “leave one subject out” on test dataset) are reported. Green bold numbers = best performance; red bold numbers = worst performance. W, Wrist; UA, Upper Arm; T, Thigh; A, Ankle; L, Lumbar Segment*.

**Table 8:** Accuracy (ACC), precision (PPV, Positive Predictive Value) and sensitivity (TPR, True Positive Rate) for each functional fitness movement, related to the cubic kernel SVM algorithm with a 600 ms length windows and 10% overlap.

		**C&J**	**AS**	**BJ**	**BP**	**TRANS**
W+A	ACC	96.4	98.4	98.8	98.0	92.5
	PPV	83.5	91.8	89.1	84.8	93.7
	TPR	79.5	74.4	76.8	80.6	96.5
W+L	ACC	96.6	98.5	98.7	97.8	92.5
	PPV	86.1	91.3	84.5	84.6	93.6
	TPR	77.7	77.4	80.8	77.5	96.6
W+T	ACC	97.3	98.1	98.7	97.9	93.0
	PPV	87.8	90.4	87.7	88.4	93.7
	TPR	84.7	69.6	77.3	75.1	97.3
W+UA	ACC	97.0	97.8	99.1	98.0	92.4
	PPV	89.5	88.1	90.9	87.6	93.0
	TPR	79.0	65.6	83.3	78.2	97.2
UA+A	ACC	95.9	98.2	99.1	98.6	92.6
	PPV	81.2	90.6	92.8	89.1	93.8
	TPR	75.4	71.1	82.3	87.7	96.6
UA+L	ACC	96.7	98.4	99.0	98.5	93.3
	PPV	88.6	93.3	89.0	89.9	93.8
	TPR	76.3	72.6	83.7	84.9	97.6
UA+T	ACC	96.7	98.2	99.1	98.5	93.3
	PPV	84.6	89.2	90.6	91.6	94.4
	TPR	80.9	73.7	85.7	83.7	96.9

*Data from pairs of IMUs were used as input. Classification performance is reported as %. For sake of clarity, only results from the most stringent validation (Stage 2 = “leave one subject out” on workout dataset) are reported. Green bold numbers = best performance; red bold numbers = worst performance. W= Wrist, UP= Upper Arm, T= Thigh, A= Ankle, L= Lumbar Segment*.

### Feature Selection Analysis

From the sensitivity analysis we identified two configurations to be further tested by using the feature selection. We considered the data collected by the sensor on the upper arm (UA configuration, for a total of 108 features) and by the combination of sensors on the upper arm and thigh (UA+T configuration, for a total of 216 features). After a qualitative analysis of the trend in prediction scores, from the most important predictor to the less significant, we set the number of the features to keep to 20.

The reduction of the number of the features did not compromise the overall accuracy of the classifier, thus underlining the reliability of the approach. In particular, the highest value of accuracy was maintained when considering the UA configuration (99.1% for BJ), whereas UA+T configuration reported a reduction of only 0.3% (99.1 vs. 98.8%) (Table 9). Furthermore, in both configurations, all the values of accuracy were >89.5% (TRANS in UA). Larger differences concerned precision and recall in classifying the AS task, which decreased to 59.3% and 49.6% (UA), and 71.9% and 62.6% (UA+T), respectively. For AS alone, both PPV and TPR decreased by 15–20%, showing risk of misclassification.

**Table 9:** Accuracy (ACC), precision (PPV, Positive Predictive Value) and sensitivity (TPR, True Positive Rate) for each functional fitness movement, related to the cubic kernel SVM algorithm with a 600 ms length windows and 10% overlap.

		**ACC (%)**	**PPV (%)**	**TPR (%)**
UA	C&J	95.4	79.6	70.8
	AS	95.8	59.3	49.6
	BJ	99.1	90.0	84.2
	BP	97.3	78.8	75.4
	TRANS	89.5	91.7	94.6
UA+T	C&J	96.0	79.3	80.5
	AS	96.9	71.9	62.6
	BJ	98.8	85.5	81.3
	BP	97.5	82.7	75.1
	TRANS	91.9	93.9	95.4

*Data from the IMU placed on the upper arm (UA) and the combination of upper arm and thigh (UA+T) were used as input. Classification performance is reported from the only Stage 2 = “leave one subject out” (LOSO) validation on test dataset. Green bold numbers = best performance; red bold numbers = worst performance in each stage*.

Most of the identified features were time-domain features (15 out of 20 for the UA configuration and 16 out of 20 for the UA+T configuration), and was information coming from gyroscope data (13 out of 20 for the UA configuration and 12 out of 20 for the UA+T configuration). In UA+T, the identified features were equally spread between the sensor placed on the upper arm and on the thigh (10 out of 20, each).

## Discussion

We developed and tested a supervised learning approach to recognizing and classifying functional fitness movements within a continuous workout, combining four different drills. Accelerations and angular velocities from a set of wearable inertia sensors were used as input of the classifier. Different machine learning algorithms, time segmentation strategies and combination of sensors were assessed. Classification accuracy was generally high in both Support Vector Machine (SVM) and k-Nearest Neighbors approaches (>82.5% in the worst case); the SVM model with cubic kernel and applied to 600 ms−10% overlap data windows gave the best performance overall (94.4–97.8% accuracy, depending on the type of validation carried out). Information coming from sensors from the upper limb, alone or in combination with a wearable unit in the lumbar area or on the thigh, appeared to be key to achieve optimal classification performance.

By using SVM on the whole dataset, misclassifications (as False Negative Rate—NFR) were lower in the “Transition” phase (0.6–2.2%) and higher in the other four drills, particularly in the “American Swing” (18.9–20.7%). *Ex post* analysis highlighted that the higher percentages of errors could be typically related to three main factors. (1) The overall smaller number of windows associated with functional movements as opposed to transitions. (2) The choice made for the “majority” criterion, whereby up to 34% of a functional movement could still belong to a window labeled as TRANS. This may have an influence on the capability of the classifier to assign a window to one of the four drills instead of TRANS. (3) The difficulty in labeling windows as belonging to a movement or TRANS between repetitions of the same exercise, when the dynamics of the task makes it difficult to establish with certainty the start and end of the movement. Combining these three items, the problem appeared more evident for the “American Swing,” possibly for the inherent dynamics of the task.

When analyzing the contribution of each sensor independently (1-sensor input) or in combination with another IMU (2-sensor input), the overall classification performance decreased of few percentage points, but still achieved an accuracy >83.2% in the worst case (i.e., IMU on the ankle, with the most stringent validation approach). Ankle kinematics may contain less information when feet are not moving; this situation may happen in a number of movement- and transition-related situations, such as during the “Clean and Jerk,” thus explaining the decreased performance of the classifier. In fact, collecting upper arm kinematics alone yielded 91.0–96.4% accuracy (depending on the validation approach). Also, adding information from a second sensor generally improved the capability of the algorithm to identify classes correctly, narrowing the performance gap between using two IMUs or the whole sensor network. The best combinations resulted from adding one further IMU to one sensor on the upper arm, i.e., upper arm and lumbar area (93.0–97.4%) or thigh (93.0–97.6%), which further confirms the need for the system to cover the widest range of movement dynamics. Similarly to what observed for the whole sensor network, misclassifications were more common in the “American Swing” (31.8–36.3% and 21.2–26.3% FNR for the UA and UA+T configurations, respectively).

To explore the translation of the selected algorithm into more easily applicable framework, a subset of features, consisting of the best 20 identified through a filter-based algorithm (mRMR), was used in a 1-sensor or 2-sensor configuration, and its classification ability tested (LOSO validation on the test dataset). The overall accuracy resulted better than 90% for all the performed task, although the confusion matrixes highlighted difficulties in distinguishing “similar” gestures (AS misclassified with TRANS). Further analysis of feature selection suggested that the most informative characteristics of the dataset were mainly related to time domain (i.e., kurtosis and skewness). Although these preliminary findings support the use of feature reduction in the pipeline of data processing, a more in-depth analysis of feature selection and outcomes derived thereof is advisable, especially for 1-sensor solutions with lower-end technology (Fan et al., 2019).

Supervised machine learning appeared a suitable tool for the automatic classification of different functional fitness exercises. Our study addressed a scenario that for number and type of movements involved appears more challenging than what has been assessed by other works in the field. Also, we located our sensors according to where existing consumer technologies would be placed, and not thinking of what the best configuration for motion capture would be. Despite these added complexities, our approach obtained similar performance to what reported by the literature as the current state of the art. Ghazali et al. (2018) achieved 91.2% accuracy in tracking several common sporting activities such as walking, sporting, jogging sprinting and jumping. Using wearable sensors and SVM/kNN methods, Mannini and Sabatini (2010) were able to distinguish between elementary physical activities such as standing, sitting, lying, walking, climbing and identify activities within sequences of sitting-standing-walking-standing-sitting with an accuracy between 97.8 and 98.3%.

Within fitness activities, Adelsberger and Troster (2013), studied 16 participants performing a squat press, and via SVM managed to detect movements with 100% accuracy and differentiate between expert and beginner performance (94% accuracy). Research on weightlifting has used different approaches, mainly aiming at recognizing the type of exercise performed (Pernek et al., 2015; Hausberger et al., 2016; O'Reilly et al., 2017), or identifying performance metrics (e.g., quality of execution, intensity, deviation from a standard pattern) for each exercise (Pernek et al., 2015; O'Reilly et al., 2017a,b,c). Approaches looking at performance metrics focus on the possibility of using personalized classifiers to monitor the quality of movement execution; they are more complex and demanding in terms of computational resources and sample sizes than what we presented in our study. On the other hand, the solutions presented in literature to address the movement recognition problem are very similar to what we have proposed. Different algorithms (such as the Random Forest—RF—and the Linear Discriminant Analysis—LDA) have been explored in the existing literature, but the overall accuracies appear comparable to the values we obtained. For the size of our dataset, SVM resulted optimal in terms of both classification accuracy and training costs. RF represented an optimal solution in multi-class problem in terms of performance and computational costs, despite requiring larger datasets (O'Reilly et al., 2017), whereas LDA was reported to perform well in simple drills classification, even allowing real-time applications, when considering a single sensor (Crema et al., 2019).

One of the main limitations of the presented work was the reduced number of involved subjects, compared to some validated machine learning approaches found in scientific literature (O'Reilly et al., 2017,a,b). Our study was exploratory, and the observed sample was relatively homogeneous in terms of sporting abilities. Having access to a larger and more varied group of participants would allow covering a wider spectrum of individual characteristics and, possibly, making the classifier more robust to inherent intra- and inter-subject variability (Preatoni et al., 2013) in movement execution. It could also allow to distinguish between expert and novice performance and/or between different level of movement intensity. Although our sample size was relatively small for typical machine learning studies, our method achieved a classification performance not inferior to equivalent approaches applied in different sports scenarios, including simple tasks, such as walking or running, and even more complex exercise including fitness training. Another potential limit lies in the labeling procedures, which relied in the use of footage from a single 50 Hz camera. A single plane of view for four distinguished movements could make establishing their exact start and finish time more difficult. Differences in sampling rates between different systems could also add minor discrepancy in time line reconstruction. Finally, a potential bias to the assessment of classification performance could be the disproportion between the periods of transition and of functional movement execution, with the former being an order of magnitude more numerous (>3,000 transition windows vs. ~100–200 windows per each functional movement). Arguably, in our application, transitions are not static, easily detectable situations, and rather contain a spectrum of movement features that are as or even more varied than the four movements of interest. Thus, high prevalence of transition intervals should not decrease the value of the solution proposed.

## Conclusions

Our study addressed a novel issue in the area of automatic activity tracking. We used wearable sensor data of the same kind of what could be provided by modern smart technologies and obtained from body locations similar to where those technologies could be secured. Classifying functional fitness movements within a continuous workout is a non-trivial task that, to the best of our knowledge, no other research had investigated. Despite the relatively small dataset used to train the algorithm, the accuracy achieved in detecting and recognizing four popular training drills was encouraging, even considering a simpler 1-sensor or 2-sensor configuration. Reducing input data to accelerations and angular velocities provided by a single sensor did not degrade excessively the classification ability of the algorithm, which still generated an overall level of accuracy similar to what obtained from the whole dataset available. These findings are particularly interesting as commercially available devices such as smart watches and/or phones contain inertial sensors and are typically worn in similar locations (i.e., upper arm and wrist) to where IMUs were attached in our study. This work perfectly fits the current technological trend on the combined use of wearable devices and artificial intelligence to track human activities automatically (Attal et al., 2015) and support sports activities (Cust et al., 2019). In the longer perspective, the proposed approach could drive the development of software and applications to aid on-field coaching and judging and provide a more objective, quantitative way to evaluate movement technique and correct/safe execution of specific drills.

## Data Availability Statement

The datasets generated for this study are available on request to the corresponding author.

## Ethics Statement

The studies involving human participants were reviewed and approved by the study was approved by the Research Ethics Approval Committee for Health (REACH) of the University of Bath, with reference number EP17/18 247. The patients/participants provided their written informed consent to participate in this study.

## Consent Statement

Written informed consent was obtained from the individuals for the publication of any potentially identifiable images or data included in this article.

## Author Contributions

Original conceptualization was designed by EP, SN, and NL. SN and EP defined the setup. SN performed the acquisition and data analysis. EP, SN, and NL contributed to the original draft preparation. EP and NL finalized the manuscript. All authors contributed to the article and approved the submitted version.

## Conflict of Interest

The authors declare that the research was conducted in the absence of any commercial or financial relationships that could be construed as a potential conflict of interest.
